# Lyophilization Reduces Aggregation of Three-Dimensional DNA Origami at High Concentrations

**DOI:** 10.1021/acsomega.3c01680

**Published:** 2023-05-11

**Authors:** Anna V. Baptist, Amelie Heuer-Jungemann

**Affiliations:** † Max Planck Institute of Biochemistry, Am Klopferspitz 18, 82152 Martinsried, Bavaria, Germany; ‡ Center for NanoScience, Ludwig-Maximilians University, 80539 Munich, Germany

## Abstract

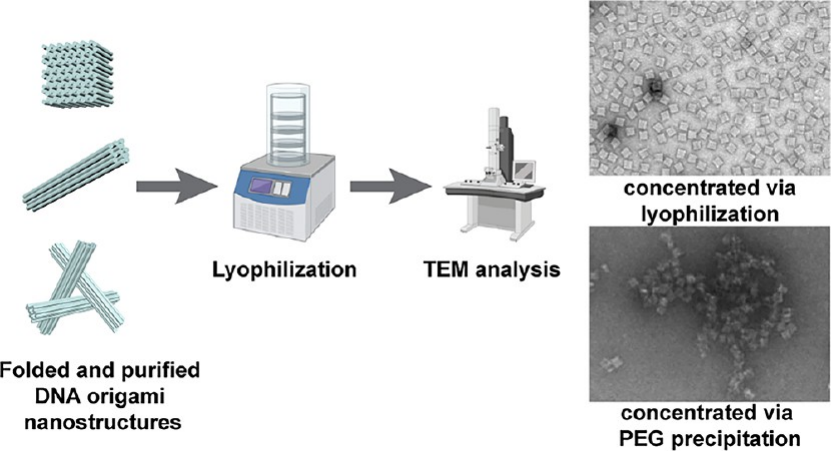

Although for many purposes, low concentrations of DNA origami are sufficient, certain applications such as cryo electron microscopy, measurements involving small-angle X-ray scattering, or *in vivo* applications require high DNA origami concentrations of >200 nM. This is achievable by ultrafiltration or polyethylene glycol precipitation but often at the expense of increasing structural aggregation due to prolonged centrifugation and final redispersion in low buffer volumes. Here, we show that lyophilization and subsequent redispersion in low buffer volumes can achieve high concentrations of DNA origami while drastically reducing aggregation due to initially very low DNA origami concentrations in low salt buffers. We demonstrate this for four structurally different types of three-dimensional DNA origami. All of these structures exhibit different aggregation behaviors at high concentrations (tip-to-tip stacking, side-to-side binding, or structural interlocking), which can be drastically reduced by dispersion in larger volumes of a low salt buffer and subsequent lyophilization. Finally, we show that this procedure can also be applied to silicified DNA origami to achieve high concentrations with low aggregation. We thus find that lyophilization is not only a tool for long-term storage of biomolecules but also an excellent way for up-concentrating while maintaining well-dispersed solutions of DNA origami.

## Introduction

The complete control over size and shape together with full site-specific addressability makes DNA origami nanostructures perfect breadboards for the arrangement of functional molecules. This has led to proposed applications ranging from nanophotonics^1,2^ to drug delivery,^3,4^ bio-sensing, and imaging.^5,6^ On the other hand, the ability to precisely control nanostructure shapes and program complex assemblies has also allowed for complex hierarchical assemblies^7^ that can reach micrometer sizes^8,9^ (two-dimensional (2D) assemblies) or the GDa scale (3D assemblies).^10^ Generally, during DNA origami synthesis, structures are folded at the 10–20 nM scaffold concentration (resulting in the same concentration of origami) in small volumes (∼100 μL). Such amounts and concentrations are sufficient for most DNA origami applications. However, for certain applications such as single-particle analysis by cryo electron microscopy (cryoEM),^11−13^ small-angle X-ray scattering (SAXS),^14−16^ for *in vivo* applications,^17−19^ or for DNA origami silicification,^16,20,21^ high concentrations (> 200 nM) are often required. Such concentrations can be achieved by various means including ultrafiltration or polyethylene glycol (PEG) precipitation.^22^ However, each of these methods has certain drawbacks: Ultrafiltration is often the method of choice for many DNA origami structures for both purification from excess staples, buffer exchange, and up-concentration.^22^ However, it only efficiently works using small filter tubes, allowing only a maximum of 500 μL of solution to be filtered at a time. For large volumes of DNA origami, this requires many filter units, which can become costly very quickly. It can also result in high losses of rod-shaped structures due to leakage or aggregation.^22,23^ PEG precipitation, on the other hand, is very cost-effective and can, in principle, be applied to any DNA origami to achieve both purification from excess staple strands as well as for up-concentration. Here, freshly folded structures at typical concentrations of 10–20 nM are mixed with a buffer containing 15% PEG, a molecular crowding agent.^22^ Centrifugation then pellets the structures and allows for redispersion in any desired volume of a buffer of choice. However, it was shown that volumes <150 μL resulted in less efficient redispersion.^22^ A major issue with both methods with respect to sample quality is the requirement for prolonged centrifugation. This can enhance aggregation due to stacking interactions or structural interlocking of certain types of 3D DNA origami. Aggregation is further enhanced by redispersion of DNA origami in low buffer volumes after centrifugation if high concentrations are required. Especially for structural analysis or *in vivo* applications, aggregates are highly undesirable and must be minimized. Aggregates may be removed by filtration but at the expense of a loss in yield and hence concentration. To overcome these issues and achieve well-dispersed DNA origami solutions with high concentrations, displaying minimized aggregation, we here introduce lyophilization as an effective tool. We show that if DNA origami structures, after purification, are kept at low concentrations in low salt buffers, subsequent lyophilization and final redispersion in ultrapure water can be utilized to achieve highly concentrated samples of 3D DNA origami with significantly reduced aggregation compared to samples up-concentrated by ultrafiltration or PEG precipitation.

Freeze-drying or lyophilization is a technique commonly employed for the long-term storage of biomolecules such as proteins, peptides, DNA, and even whole mammalian or bacterial cells.^24^ In this technique, a liquid sample is shock-frozen in liquid nitrogen followed by sublimation of the liquid under vacuum, leaving behind only solid components. Lyophilization is a gentle technique as molecules go straight from the solid frozen to the solid dry state, without an intermediate solution step. Lyophilization has also been proposed as a long-term storage option for DNA nanostructures, including 2D DNA origami and tetrahedral nanostructures.^25,26^ However, the effect of lyophilization on 3D DNA origami and especially its effect on DNA origami aggregation has thus far not been explored. Here, we exemplify the advantage of using lyophilization for up-concentration of 3D DNA origami with reduced aggregation by using different designs (14- and 24-helix bundles (HBs), a four-layer block (4LB), a cube, and a tensegrity triangle (SC), see Figures S1–S5 for CaDNAno designs), which all display different aggregation behaviors including tip-to-tip stacking, side-to-side binding, or structural interlocking at high concentrations. These types of aggregates can be significantly reduced by lyophilization. It should be noted that the types of aggregates that already occur during folding, e.g., complete or partial misfolding or structures sharing one scaffold, remain unaffected and cannot be reduced by this technique.

During lyophilization, not only the DNA origami but also buffer components, including salts, are left as solid residues, and structures must initially be dispersed in a low salt buffer so as to avoid exceedingly high salt concentrations after lyophilization and redispersion in small volumes of water. We show that in the absence of EDTA, structures are stable for at least 4 weeks in low salt buffers (as low as 50 μM MgCl_2_) at room temperature, confirming recent results by Keller and co-workers.^27^ However, we also found that tightly packed structures required slightly more salt for prolonged structural integrity. Starting with an initially low concentration of DNA origami results in very well-dispersed structures with minimal interaction. This is maintained during shock-freezing and subsequent lyophilization, resulting in very low amounts of aggregation, even after redispersion in low buffer volumes to achieve high concentrations. Finally, we show that not only bare DNA origami but also silicified structures can be easily and gently up-concentrated by lyophilization. We thus establish the freeze-drying process as a very efficient way to achieve high concentrations of bare and silicified DNA origami with excellent storage capabilities and significantly reduced aggregation.

## Materials and Methods

### DNA Origami Design, Synthesis, and Purification

DNA origami nanostructures were assembled from the p8064 (Tilibit Nanosystems) or the p8634 scaffold (produced in-house) and the respective staple strands (IDT) in 1× Tris-acetate-EDTA (TAE) buffer containing 18 or 20 mM MgCl_2_ using a temperature ramp, as described previously.^15,16^ The folded DNA origami nanostructures were purified either via ultrafiltration with a 100 kDa cutoff (Amicon filter units) or via PEG precipitation.^22^ For Amicon ultrafiltration, 500 μL of the folding solution was applied to each pre-wetted filter unit and centrifuged at 8000 rcf for 8 min. After centrifugation, the flow-through was discarded and the filter units were refilled with 400 μL of the respective buffer. This procedure was repeated four times. After the elution, ultrafiltration was repeated with new filter units and at least three more washing steps. For PEG precipitation, 1 mL of EDTA-free PEG buffer containing 15% (w/v) of PEG-8000, 1× TA, and 500 mM NaCl was added to 1 mL of folding solution and the MgCl_2_ content was adjusted to 10 mM MgCl_2_. After careful mixing, the solution was centrifuged at 20,000 rcf for 30 min. The supernatant was discarded immediately after centrifugation, and the pellet was resuspended in at least 500 μL of buffer containing 500 μM or 2.5 mM MgCl_2_, depending on the respective DNA origami structure. The sample was then placed on a shaker at 500 rpm at 30 °C for at least 16 h. For lyophilization, samples were diluted to 10 nM DNA origami concentration using the respective low salt buffer immediately after purification. For strongly concentrated samples (no lyophilization), buffers containing 10 mM MgCl_2_ were used for resuspension to reach final origami concentrations of 200–250 nM. PEG precipitation was employed for all DNA origami nanostructures in this study. Optionally, ultrafiltration was used for the 24HB and the SC only.

### DNA Origami Silicification

Implementing our previously established protocol,^16^ DNA origami solutions were used at a concentration of 200 nM in a total reaction volume of 50 μL and were dispersed in 1× TAE buffer containing 3 mM MgCl_2_. After placing the samples on a thermo shaker, the first silica precursor *N*-trimethoxysilylpropyl-*N*,*N*,*N*-trimethylammonium chloride (TMAPS) (TCI, diluted 1:19 in methanol) was added to the sample in 5-fold molar excess to the number of nucleobases. The samples were then left shaking on the thermo shaker for 1 min (300 rpm, 21 °C) before the second silica precursor tetraethyl orthosilicate (TEOS) (Sigma-Aldrich, diluted 1:9 in methanol) was added to the samples in 12.5-fold molar excess to the number of nucleobases. After another 15 min of shaking on the thermo shaker, the samples were transferred to a tube revolver rotator (Thermo Fisher Scientific) and rotated at 40 rpm at 21 °C for 4 h. Subsequently, one round of ultrafiltration (Amicon filter units, 30 kDa cutoff) was performed on the samples for purification from excess silica precursors.

### Lyophilization

For the lyophilization procedure, up to 750 μL of the DNA origami sample dispersed in low salt buffer at a concentration of around 10 nM was placed in a 2 mL Eppendorf tube. The lid of the tube was opened, and a second lid with several small holes was placed on top of the tube. The sample was shock-frozen in liquid nitrogen and placed into a pre-cooled Eppendorf rack. The samples were immediately transferred to the lyophilizer (Lyovac) and lyophilized overnight. After the lyophilization procedure, samples were either placed on the lab bench for storage at room temperature or directly resuspended in ultrapure water to reach a concentration of about 200–250 nM. N.B.: as all buffer components, including salt, are retained in the sample, redispersion in ultrapure water is sufficient.

### Agarose Gel Electrophoresis

All DNA origami samples and the scaffolds used as a reference were diluted to a concentration of 10 nM prior to the addition of Ficoll loading buffer containing orange G. Then, all samples were applied onto 0.7/2% (w/v) agarose gels containing 11 mM MgCl_2_ and 1× TAE buffer. The gels were run in 1× TAE buffer with 11 mM MgCl_2_ at 75 V for 90–120 min at 4 °C.

### Agarose Gel Analysis

To determine the relative amount of monomers in a sample, the brightness of the monomer bands and of the entire gel lane including the (aggregates in the) wells was analyzed. The regions of interest were selected by drawing a rectangular box around them using the ImageJ software^28^ and the integrated densities (intensities) of the selected areas and of the gel background without any sample were extracted. After background subtraction, the values for the monomer bands alone and the corresponding gel lane were compared for all DNA origami nanostructures tested. Three different gels of three different lyophilization experiments were used for the analysis of each structure.

### TEM Imaging

TEM imaging was carried out on a Jeol-JEM-1230 TEM operating at an acceleration voltage of 80 kV. For sample preparation, 10 μL of DNA origami samples diluted to 5 nM immediately before TEM grid preparation was applied to plasma-cleaned TEM grids (formvar/carbon-coated, 300 mesh Cu, TED Pella Inc.) for 5 min. After careful removal of the sample liquid with a filter paper, the grid was washed with 5 μL of a 2% uranyl formate solution. Subsequently, the uranyl formate solution was applied to the grid for 45 s for staining purposes (for bare samples). Grids with silicified samples were instead washed once with 5 μL of ultrapure water. After removal of the excess liquid, the grid was left for air-drying and subsequent imaging.

## Results and Discussion

**Figure 1:**
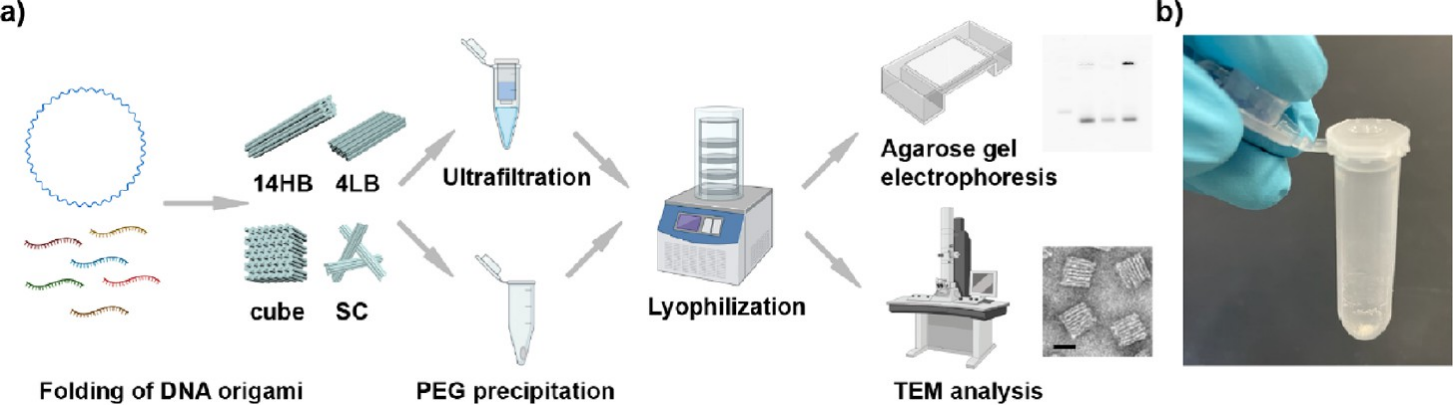
Overview of the workflow for the lyophilization of 3D DNA origami nanostructures. (a) Scheme depicting the general procedure from DNA origami folding to structural analysis after lyophilization. (b) Image of the lyophilized white powder (SC) directly after lyophilization and of the 2 mL Eppendorf tube with the perforated lid.

For several applications, high concentrations of DNA origami solutions are required. However, commonly applied up-concentration procedures often also result in aggregation. In low concentrations, however, aggregation is often not a problem. We therefore hypothesized that aggregation could be reduced if structures were directly transferred from a well-dispersed state at a low concentration to a solid state without slow volume reduction or centrifugation. A method that allows for this is lyophilization. Figure 1a depicts the general workflow of our lyophilization procedure. After folding of the different DNA origami nanostructures (14HB, 24HB, 4LB, cube, and SC), the samples are purified from excess staple strands by ultrafiltration or PEG precipitation, dispersed in larger volumes of low salt buffers to achieve low origami concentrations and subsequently lyophilized overnight before resuspension and analysis by transmission electron microscopy (TEM) or agarose gel electrophoresis (AGE).

**Figure 2:**
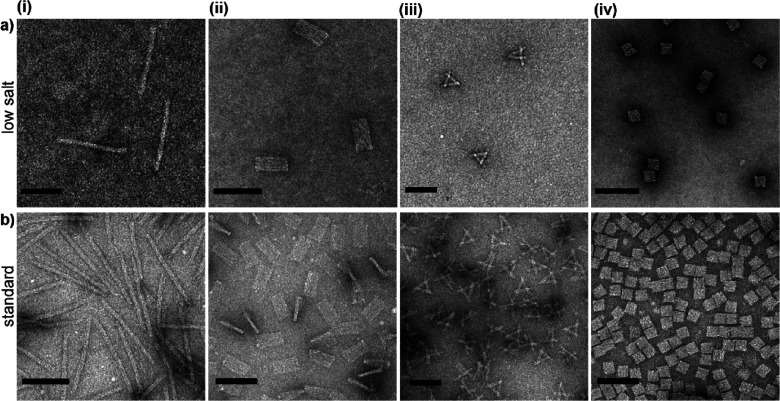
Comparison of TEM images for different 3D DNA origami nanostructures in a (a) low salt buffer (500 μM MgCl_2_ for 14HB, 4LB, and SC or 2.5 mM MgCl_2_ for cube) and in a (b) “standard” buffer (10 mM MgCl_2_). (i) 14HB, (ii) 4LB, (iii) SC, and (iv) cube. Scale bars are 100 nm.

The most commonly used DNA origami purification methods involve ultrafiltration in centrifugal filter units, PEG precipitation, size exclusion chromatography (SEC), AGE, or the use of different types of solid support such as magnetic beads.^22,29^ However, these methods have different benefits and disadvantages and are not equally well suited for different shapes and types of DNA origami nanostructures. For instance, ultrafiltration is not well suited for the purification of thin, rod-like DNA origami such as the 14HB and 24HB as high losses can occur due to DNA origami rods getting stuck in the filter membrane. AGE, SEC, and magnetic bead purification are excellent for achieving samples of high purity but are unsuitable for obtaining high concentrations of DNA origami. Therefore, we here employed PEG precipitation and ultrafiltration as high concentrations of DNA origami can be reached using these techniques. However, due to ease of use, compatibility with all DNA origami structures, and cost-effectiveness, PEG precipitation was preferably used. Samples were initially folded at 10 nM scaffold concentration and subsequently purified by PEG precipitation. The resulting DNA origami pellets were then resuspended in large volumes (>500 μL, depending on the initial folding volume and initial scaffold concentration) of a low salt buffer (50 μM to 2.5 mM MgCl_2_) and subsequently placed on a thermo shaker at elevated temperatures (∼30 °C) to ensure a proper dissolution of the DNA origami pellet and achieve a well-dispersed sample with relatively low concentrations (10 to a maximum of 30 nM). The choice of buffer in which the DNA origami is dispersed is of great significance for the subsequent lyophilization procedure. As all salts and buffer components remain in the sample during and after lyophilization, an increase in the concentration of the DNA origami by resuspension of the lyophilized powder in a significantly lower liquid volume than in the original sample before lyophilization will also inevitably lead to a significantly increased concentration of the salts (here, usually mostly MgCl_2_). This can lead to enhanced aggregation. An initial buffer or aqueous solution with a low MgCl_2_ content is therefore highly recommendable since it also aids to decrease the potential of DNA origami aggregation due to reduced electrostatic screening. Importantly, the initial amount of MgCl_2_ in the sample solution must be chosen so that stability and structural integrity of the DNA origami are not negatively affected. Kielar et al. recently showed that 2D DNA origami structures were stable at very low concentrations of Mg^2+^ or even in pure water in the absence of EDTA.^27^ However, for 3D structures, this had not been thoroughly tested. We therefore investigated the stability of all four different 3D DNA origami nanostructures employed here as well as their tendency to aggregate before and after the lyophilization procedure in a range of different MgCl_2_ concentrations (see Figure S6 for a representative gel of the 14HB). All structures, apart from the cube, were found to be stable in low Mg^2+^ solutions (50–500 μM) as can be seen from TEM analysis shown in Figure 2. However, the cube, being the most tightly packed structure, lost its structural integrity very quickly after dispersion in a 500 μM MgCl_2_ solution (see Figure S7). Therefore, a higher salt content of 2.5 mM MgCl_2_ was chosen for this nanostructure (see Figure 2). For all DNA origami, we also tested the long-term stability in the low salt buffer chosen for the lyophilization experiments. For this, samples were stored on a bench in ambient conditions for up to 4 weeks (see Figure S8). All structures displayed excellent long-term stability in the low salt buffer, suggesting that solutions of purified origami could also easily be stored under ambient conditions before up-concentration by lyophilization. It should be noted that when using buffers with a low MgCl_2_ content (<1 mM), the addition of EDTA or acetic acid should be avoided as these can reduce the effective amount of MgCl_2_ available for DNA origami stabilization.^30^ As such, we found no detrimental effect of pure Tris buffer (10 mM) on the structural integrity of the DNA origami structures, whereas the use of 1× Tris acetate (TA) buffer led to the disintegration of all structures at a low MgCl_2_ content (see Figure S9).

After establishing the stability of all structures in the required low salt buffer, samples were prepared for lyophilization. For this, DNA origami samples were prepared at 10 nM concentration in up to 750 μL low salt buffer in 2 mL round-bottom Eppendorf tubes. These were fitted with an additional lid that was perforated with a syringe needle to contain several (8–10) small holes (see Figure 1b). Subsequently, the samples were immediately shock-frozen in liquid nitrogen. We found shock-freezing to be more effective than slower freezing at −80 °C as some structures could not be efficiently lyophilized in this condition (i.e., incomplete sublimation (data not shown)). After shock-freezing, samples are then directly transferred to the lyophilizer and freeze-dried overnight. For resuspension of the freeze-dried powder, ultrapure water was added to each lyophilized sample either directly after lyophilization or after a few days of storage at room temperature (see Figure S10) followed by gentle shaking for several hours on a thermo shaker. Importantly, the volume of ultrapure water used for resuspension should be at least 25–30 μL to ensure proper dissolution of the whole sample. The most important experimental parameters for the lyophilization procedure are summarized in Table S1.

**Figure 3:**
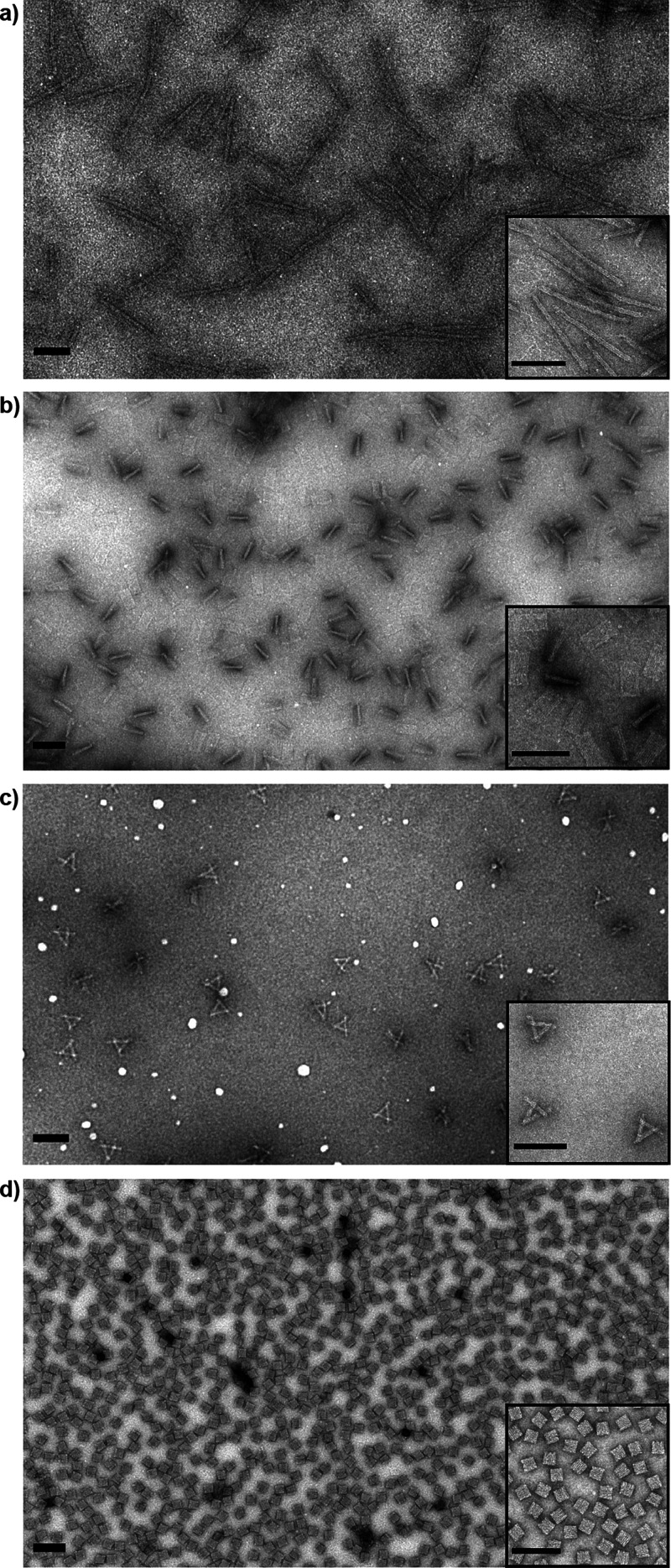
TEM images of lyophilized 3D DNA origami nanostructures: (a) 14HB, (b) 4LB, (c) SC, and (d) cube. Scale bars are 100 nm.

Although lyophilization is a well-established technique for the preservation of many biomolecules and has successfully been tested for 2D DNA origami,^25^ it was found that tetrahedral DNA nanostructures, depending on the Mg^2+^ content of the buffer, exhibited a reduced quality after lyophilization and redispersion.^26^ Moreover, the effect of lyophilization on the structural integrity of 3D DNA origami is thus far unknown. The procedure involves two stresses, freezing and drying, that could possibly be damaging to the DNA origami. Although it was previously established that DNA origami can withstand several freeze–thaw cycles without loss of structural integrity,^31^ we analyzed each structure after lyophilization and dissolution in water by TEM and AGE. Encouragingly, as can be seen from Figure 3, all structures remained intact and well dispersed, suggesting that they were not negatively affected by the procedure. Additionally, samples stored for several days under ambient conditions directly after lyophilization equally appeared structurally intact and well dispersed by TEM and AGE even at very high concentrations (>200 nM; see Figures S10 and S11). This suggests that lyophilized DNA origami can be stored in powder form without any observable detrimental effects.

**Figure 4:**
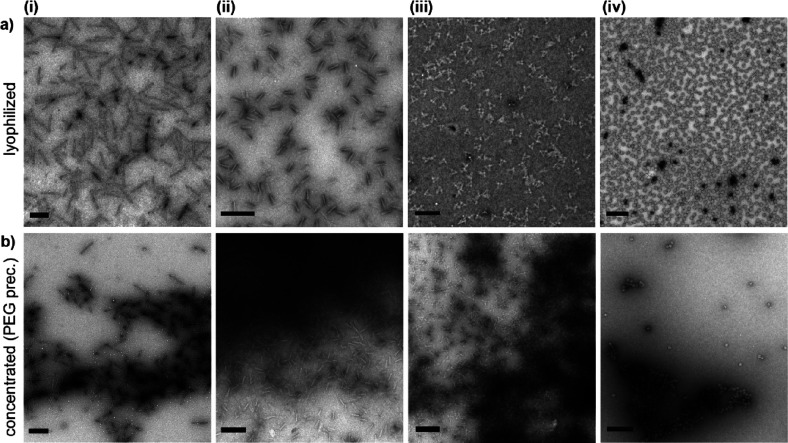
Representative TEM images of (a) different DNA origami up-concentrated (∼200 nM) via lyophilization and subsequent resuspension in a low volume of ultrapure water and (b) samples up-concentrated to the same concentration using PEG precipitation, clearly showing a higher degree of aggregation in PEG up-concentrated samples. (i) 14 HB, (ii) 4LB, (iii) SC, and (iv) cube. Scale bars are 200 nm.

**Figure 5:**
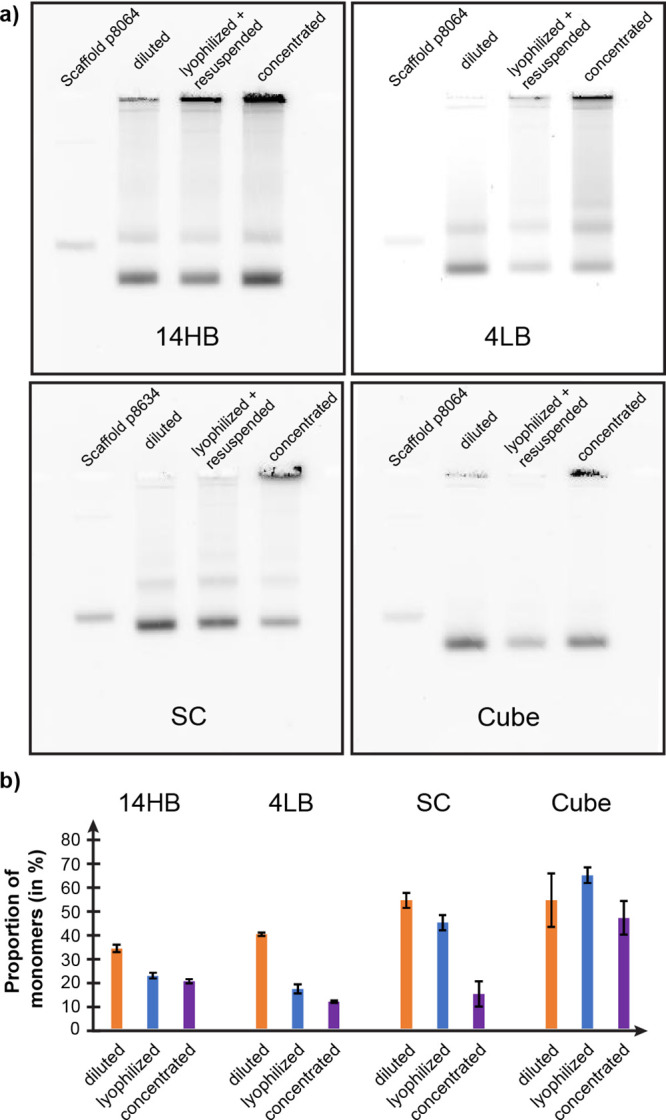
(a) Representative images from agarose gel electrophoresis for different DNA origami nanostructures comparing diluted samples and strongly concentrated samples after lyophilization and after PEG precipitation (all diluted to ∼10 nM DNA origami concentration, dispersed in an aqueous solution containing 10 mM MgCl_2_). (b) Bar chart depicting the proportion of monomers in the diluted, lyophilized, and concentrated samples for different types of 3D DNA origami, derived from gel images. Error bars represent the standard deviation (*n* = 3).

After ensuring that the structures were not negatively affected by lyophilization, we next investigated if the procedure could be used for up-concentration while reducing aggregation. Conventional methods used for up-concentration (i.e., ultrafiltration or PEG precipitation (or combinations thereof)) rely on centrifugation and/or volume reduction often leading to aggregation and subsequently bad sample quality (see Figures 4 and 5 and Figure S12). Types of aggregation commonly observed in 3D DNA origami are tip-to-tip stacking or side-to-side binding (see Figure S13). Tip-to-tip stacking can be reduced by adding either single-stranded scaffold loops to the tips of the nanostructures/leaving out the end staples during DNA origami folding or adding poly-T or poly-C tails as well as dispersion in low Mg^2+^ buffers.^22^ However, although such strategies were employed for the DNA origami structures used in this study, aggregate formation due to tip-to-tip stacking at high concentrations was still observable. Another potential cause for DNA origami aggregation/clustering is structural interlocking. This will most likely occur in nanostructures with complex 3D shapes such as the SC in this study, where struts of one structure get tangled up in another structure. Nevertheless, most of these aggregation behaviors can be minimized and almost completely avoided by keeping DNA origami at low concentrations and in low salt buffers.^22^ Thus, after synthesis and purification, we redispersed the origami in larger volumes of low salt buffer achieving DNA origami concentrations of only ∼10 to a maximum of 30 nM before lyophilization. Shock-freezing subsequently keeps structures in this “well-dispersed” (now frozen) state. As the lyophilization technique is based on sublimation, frozen structures are directly transferred from the frozen solid state (no interactions possible) to the dry solid state (no significant interactions possible). Subsequent redispersion then results in the maintenance of this well-dispersed state, even at high concentrations. Losses during lyophilization are generally very small; therefore, the required amount of water for redispersion of the origami to a desired final concentration can be easily back-calculated from the initial origami concentration and solution volume. Similarly, the final MgCl_2_ concentration in the redispersed sample can be determined. Therefore, dissolution of the DNA origami powder in low volumes of ultrapure water can result in very high concentrations of DNA origami. In our experiments, DNA origami concentrations in a range of 200–250 nM could be easily achieved from 10 nM starting concentrations.

Analysis of highly concentrated samples after lyophilization revealed a significantly increased amount of well-dispersed DNA origami monomers in strongly concentrated samples (∼200 nM) compared to samples obtained at a similar concentration by PEG precipitation (see Figures 4 and 5 and Figures S14 and S15), confirming our hypothesis that lyophilization can indeed reduce aggregation in highly concentrated samples. We analyzed and compared samples of all DNA origami structures up-concentrated by the two different methods using TEM (Figure 4) and AGE (Figure 5). As can be seen in Figure 4a, structures appear well dispersed with minimal amounts of small aggregates after lyophilization. On the other hand, samples up-concentrated by PEG precipitation (Figure 4b) showed significantly higher amounts of both larger and smaller aggregates. This trend can also be observed by AGE, where lyophilized samples displayed lower amounts of aggregates in the gel pockets (Figure 5a).

Although the magnitude of the positive effects of the lyophilization procedure was observed to vary with DNA origami shape, we found that the overall amount and the average size of aggregates were significantly reduced in the lyophilized samples (see Figures S14 and S15). The most pronounced improvement of sample quality could be observed for the SC and cube origami structures (see Figures 4 and 5), where the relative amount of DNA origami monomers in the strongly concentrated but lyophilized samples was similar to those in the diluted samples (before lyophilization). In both cases, the TEM images recorded from the lyophilized samples show a vast majority of well-distributed intact monomers and only very few small aggregates. For the 4LB, the sample quality could also be improved including a ∼1.5-fold increase in the proportion of monomers and a reduction of very large (>1 μm) aggregates (see Figure 4 and Figures S14 and S15). For the 14HB origami, already the diluted sample exhibited a stronger tendency to aggregate in the agarose gel compared to other structures, and the amount of monomers in the strongly concentrated samples appears to have been only slightly increased by lyophilization (analysis of agarose gels; Figure 5b). However, the TEM images surprisingly showed a significantly reduced average size of the aggregates and an almost complete elimination of extremely large 14HB aggregates (see Figure S14). Additionally, it has to be noted that the aggregation for 14HB samples worsened with increasing MgCl_2_ concentration both before and after lyophilization (see Figure S6).

**Figure 6:**
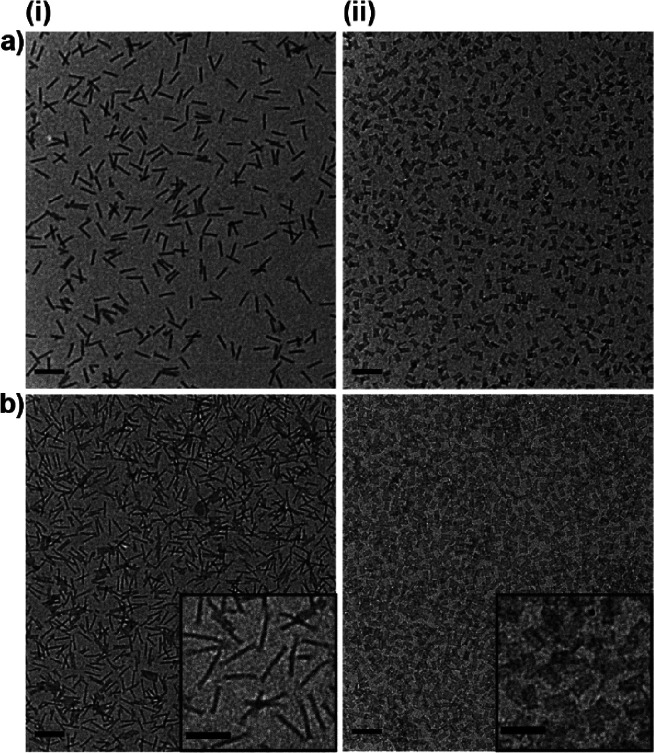
TEM images of silicified DNA origami before (a) and after (b) lyophilization and subsequent resuspension in MilliQ water for the 24HB (i) and the 4LB (ii). Scale bars are 200 nm (large images) and 100 nm (insets).

Finally, we also tested our lyophilization procedure on silicified DNA origami samples. DNA origami-templated silica nanostructures have recently gained more and more attention due to their excellent combination of properties of both the DNA origami and the inorganic silica component.^16,20,21,32−37^ They could present excellent candidates for *in vivo* applications due to their high stability and excellent biocompatibility. Therefore, the ability to form such structures and store them at high concentrations without an onset of aggregation is highly desirable. We employed our previously established silicification protocol^16,20,21^ to 24HB and 4LB samples including a purification step after the silicification. The purified samples were diluted to 10 nM in ultrapure water, shock-frozen in liquid nitrogen, lyophilized, and resuspended to high concentrations as before. TEM analysis after lyophilization/resuspension clearly demonstrates that silicified structures, not only maintained their structural integrity but also appeared to be well dispersed. Although the concentration of the samples was again significantly increased after lyophilization compared to the diluted state after purification, the silicified samples did not exhibit a strong tendency to aggregate (see Figure 6). As silicification protects the DNA origami against detrimental influences such as heat or degradation by DNases as well as increasing their stability in low salt environments, the use of a low salt buffer or ultrapure water for lyophilization experiments is unproblematic in this case. Nevertheless, the handling and storage of silicified DNA origami after the silicification reaction and particularly their up-concentration require extra caution to avoid clustering and aggregation (see Figure S16). We have shown here that the lyophilization procedure can alleviate these problems.

## Conclusions

In conclusion, we present a procedure for the up-concentration of 3D DNA origami to concentrations >200 nM via lyophilization, applicable to both bare and silicified nanostructures, that can contribute to a significant improvement of the sample quality by reducing aggregation in strongly concentrated DNA origami solutions. For all tested types and shapes of 3D DNA origami, their structural integrity was very well preserved after dispersion in a solution with a very low MgCl_2_ content (500 μM or 2.5 mM, depending on the specific DNA origami), lyophilization, and resuspension in ultrapure water. Additionally, TEM and AGE analyses of strongly concentrated DNA origami samples showed an increased proportion of well-dispersed monomers and/or a decreased number of very large aggregates in the lyophilized samples. This positive effect was found to be dependent on the shape of the 3D DNA origami. Especially for more complex 3D DNA origami shapes (SC and cube), the sample quality could be significantly improved using this up-concentration method compared to conventional up-concentration methods such as PEG precipitation.

We attribute the reduction in DNA origami aggregation to the following three main points: (i) the use of low initial DNA origami concentrations dispersed in a buffer with a low MgCl_2_ content; (ii) the avoidance of intense centrifugation during the concentration procedure; and (iii) the sublimation procedure allowing structures to go from a well-dispersed solution to a frozen solid and finally to a dry solid state, hindering excessive detrimental interactions between structures. Aggregation behaviors such as tip-to-tip stacking, side-to-side binding, and structural interlocking can be significantly reduced by lyophilization/redispersion. Nevertheless, it has to be noted that other types of aggregation caused by scaffold sharing of two monomers or other types of misfolding cannot effectively be reduced by lyophilization but must be improved through design and folding optimizations. However, aggregation caused by defective structures with unfolded scaffold sections that entangle correctly folded monomers can potentially be marginally improved by initial dispersion at a low concentration and subsequent lyophilization.

We anticipate that our findings can significantly facilitate sample preparation and improve sample quality for applications where very high DNA origami concentrations above 200 nM are required. Furthermore, the lyophilization procedure can also be applied to silicified DNA origami. Storage of bare or silicified structures as solid powder without the need for cooling or freezing furthermore enables more sustainable transport possibilities. All in all, we establish lyophilization as an excellent tool for the preparation of well-dispersed (silicified) DNA origami solutions with high concentrations.

## Supporting Information Available

CaDNAno design files of the studied DNA origami nanostructures, additional TEM and agarose gel images, and a table summarizing important experimental parameters for the lyophilization procedure (PDF)
