# Supplementary material for: Lyophilization Reduces Aggregation of Three-Dimensional DNA Origami at High Concentrations
Source: ACS Omega. 2023 May 11;8(20):18225–33. doi: 10.1021/acsomega.3c01680 (PMC10210204; doi:10.1021/acsomega.3c01680)
Supplement: Supplementary file 1 — ao3c01680_si_001.pdf [file ao3c01680_si_001.pdf]

Supporting Information

Lyophilization reduces aggregation of 3D DNA origami at high concentrations

Anna V. Baptist[1] and Amelie Heuer-Jungemann[1,2,]*

[1]Max Planck Institute of Biochemistry, Am Klopferspitz 18, 82152 Martinsried, Germany

[2]Center for Nanoscience, Ludwig-Maximilians University, Munich, Germany

1. **DNA origami designs: CaDNAno files**
2. **Testing of buffers with different $MgCl_2$ content for lyophilization**
3. **Stability of different bare 3D DNA origami in low salt buffers**
4. **Storage of lyophilized 3D DNA origami nanostructures**
5. **Summary of important parameters for the lyophilization experiments on 3D DNA origami**
6. **Comparison of different methods for purification and concentration of DNA origami**
7. **Different types of aggregation for 3D DNA origami**
8. **Additional TEM images for different up-concentrated DNA origami nanostructures**

9. TEM images for silicified DNA origami nanostructures after up-concentration via ultrafiltration

Note S1: DNA origami designs

DNA origami nanostructures were designed using the caDNAno[1] software (design schematics in Figures S1-S5).

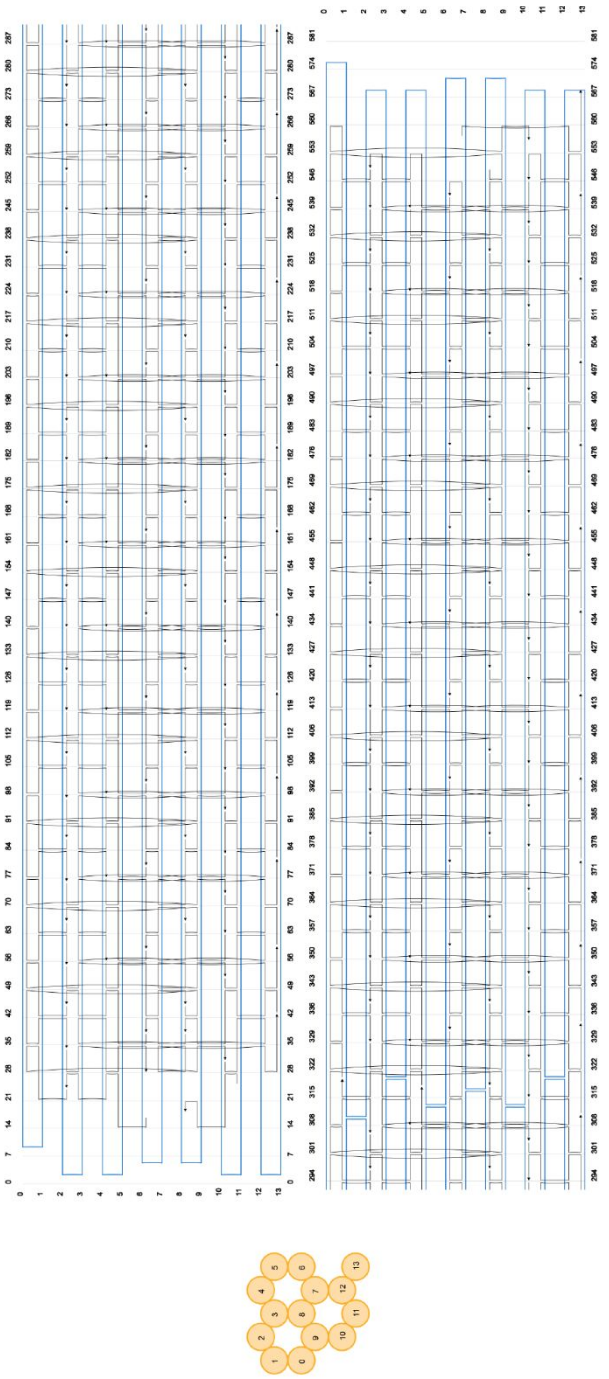

Figure S1: CaDNAno layout of scaffold (blue, p8064) and staple paths (black) of the 14-helix bundle (14HB) DNA origami structure. End staples were left out to reduce tip-to-tip stacking of 14HBs.

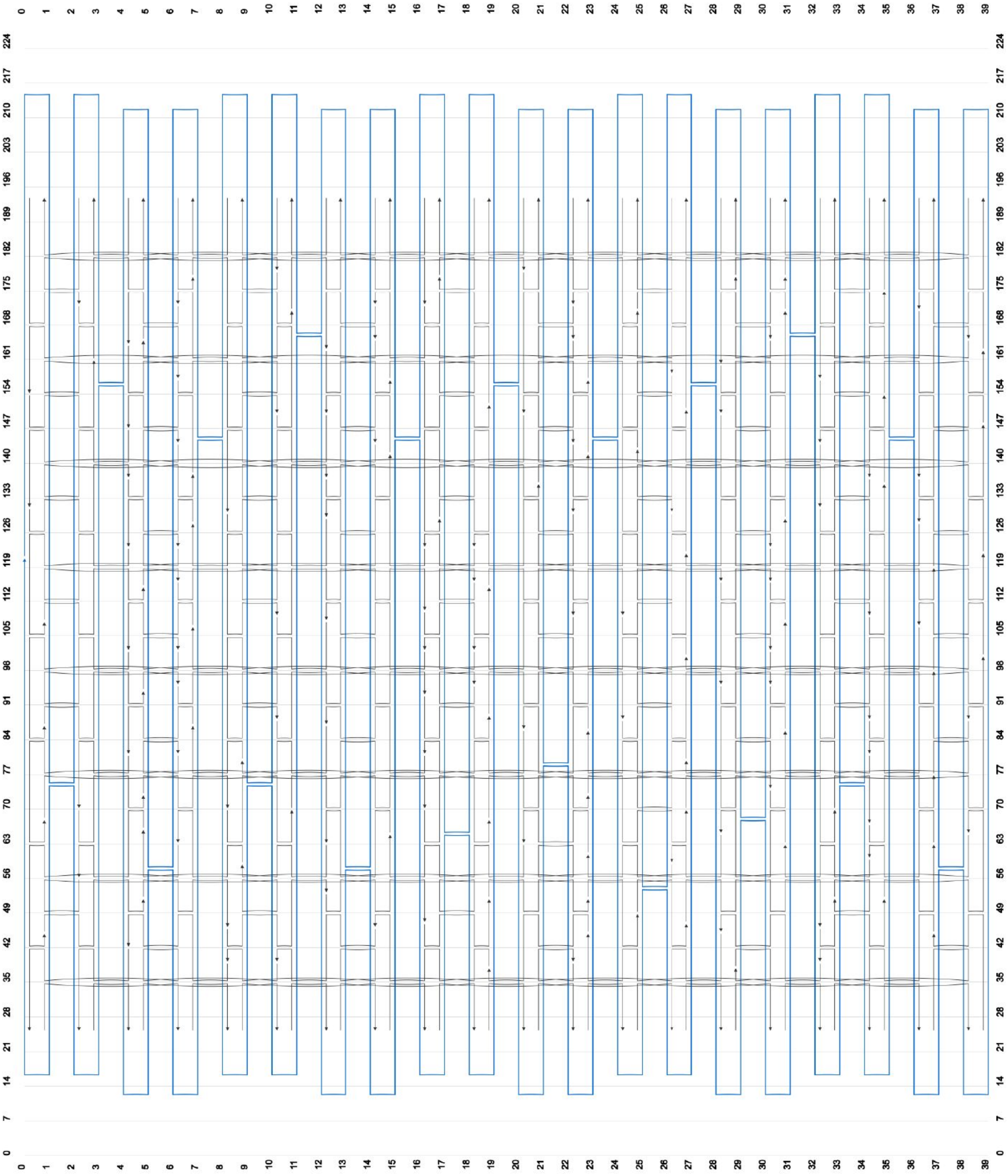

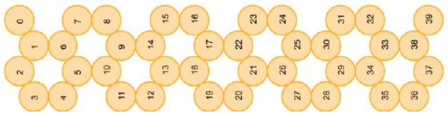

Figure S2: CaDNAno layout of scaffold (blue, p8064) and staple paths (black) of the four-layer block (4LB) DNA origami structure. The scaffold loops at the tips are supposed to reduce tip-to-tip-stacking among 4LBs.

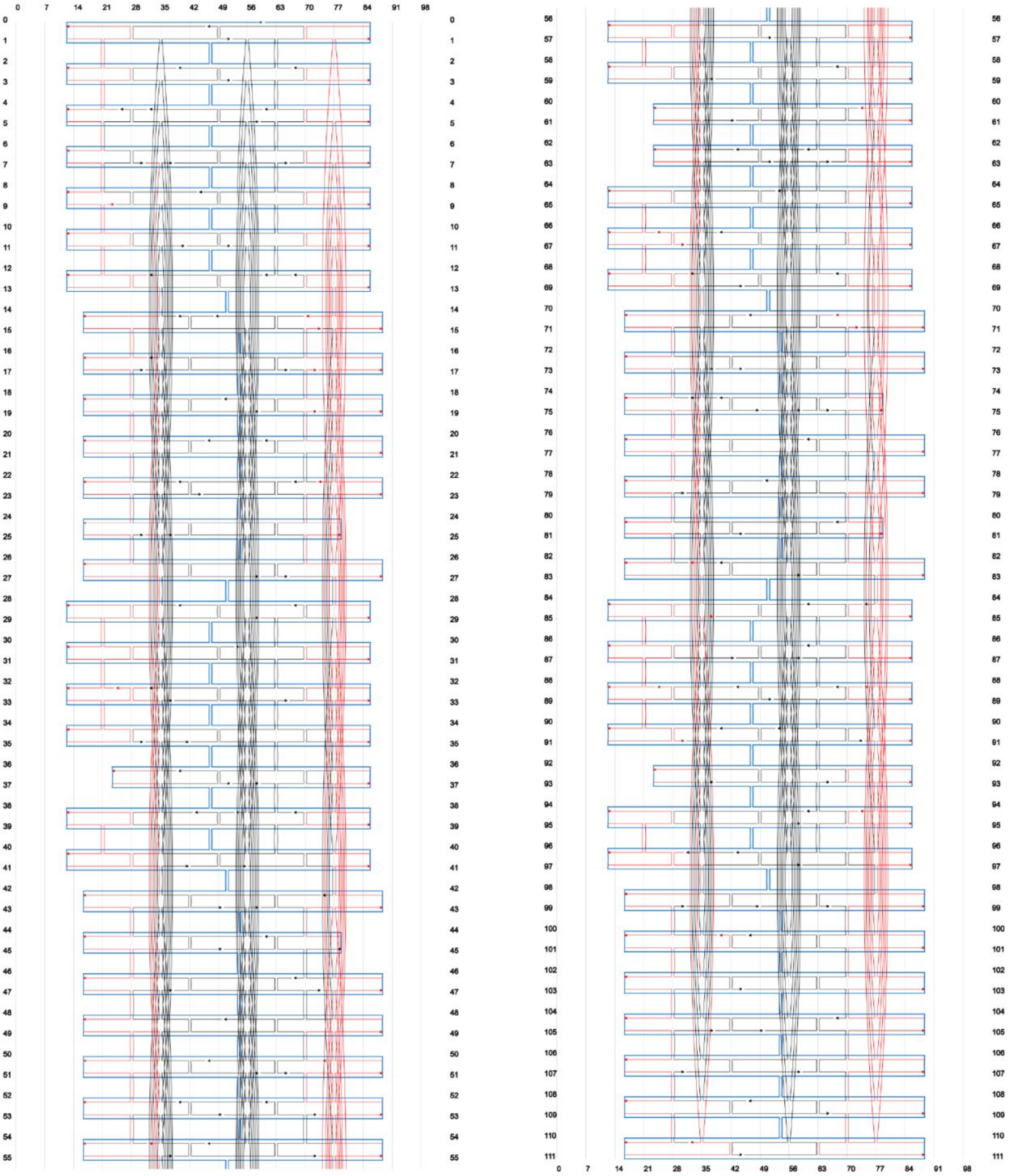
0 7 14 21 28 35 42 49 56 63 70 77 84 91 98

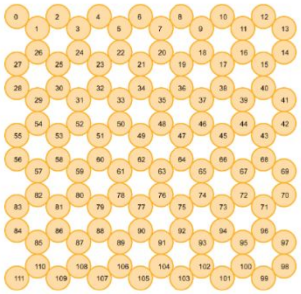

Figure S3: CaDNAno layout of scaffold (blue, p8064) and staple paths (black: core staples; red: end staples) of the cube DNA origami structure. End staples with poly-T tails were used to reduce tip-to-tip stacking among cubes.

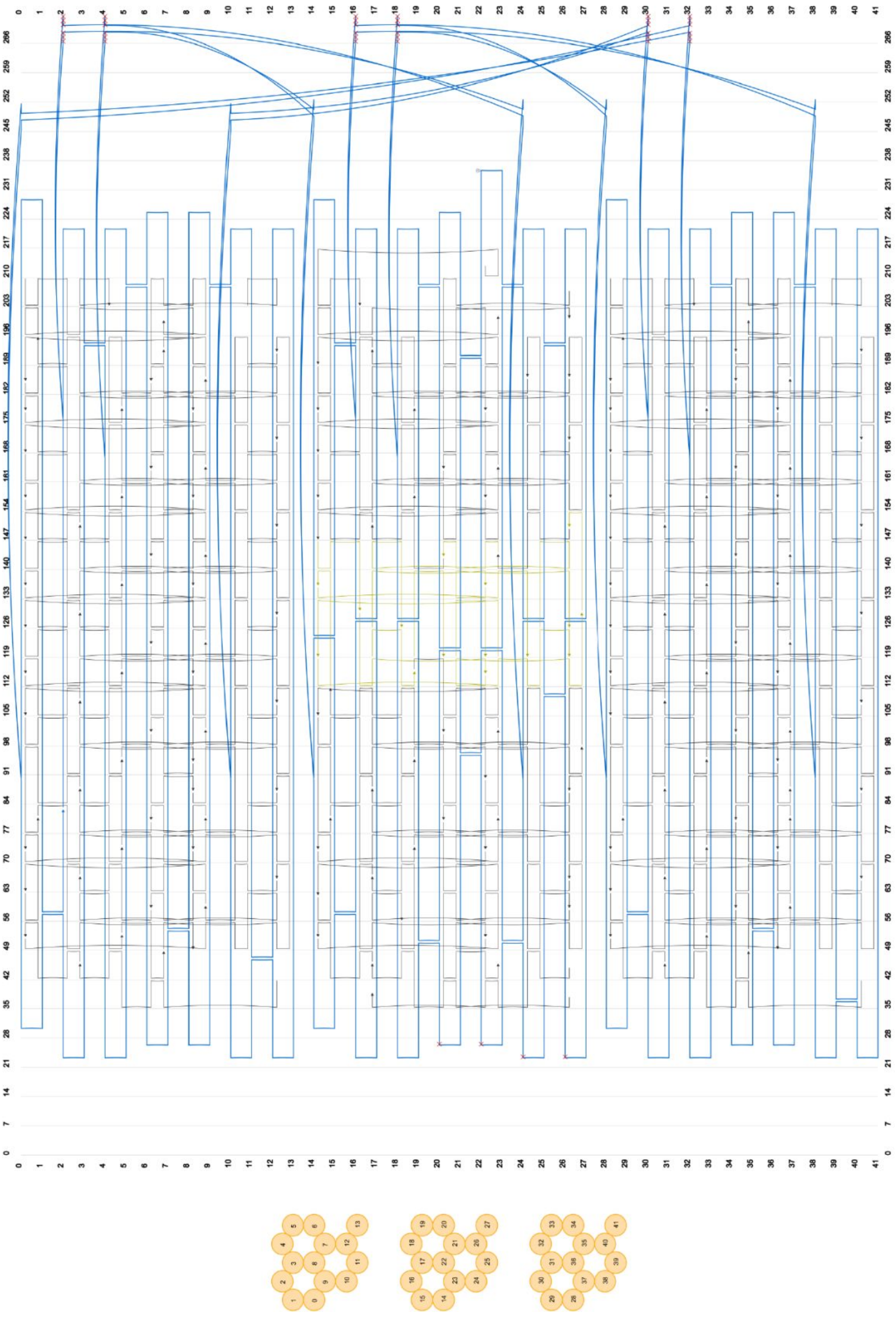

Figure S4: CaDNAno layout of scaffold (blue, p8634) and staple paths (black, yellow) of the tensegrity triangle (SC) DNA origami structure. The yellow staples are the seam staples of the second strut which are added later during the folding procedure to ensure the correct formation/assembly of the tensegrity

triangle structure.[2] The end staples at the tips of the struts were left out to avoid tip-to-tip stacking of the DNA origami monomers.

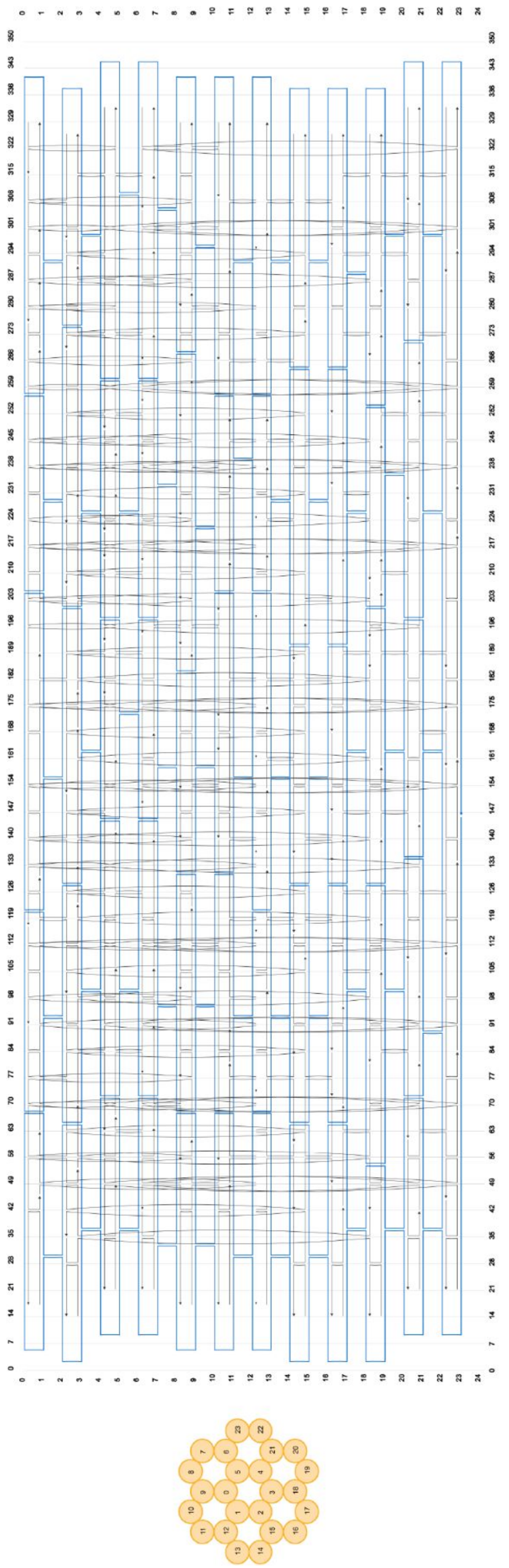

Figure S5: CaDNAno layout of scaffold (blue, p8064) and staple paths (black) of the 24-helix bundle (24HB) DNA origami structure. The scaffold loops at the tips are supposed to reduce tip-to-tip-stacking among 24HBs.

Note S2: Testing of buffers with different $MgCl_2$ content for lyophilization

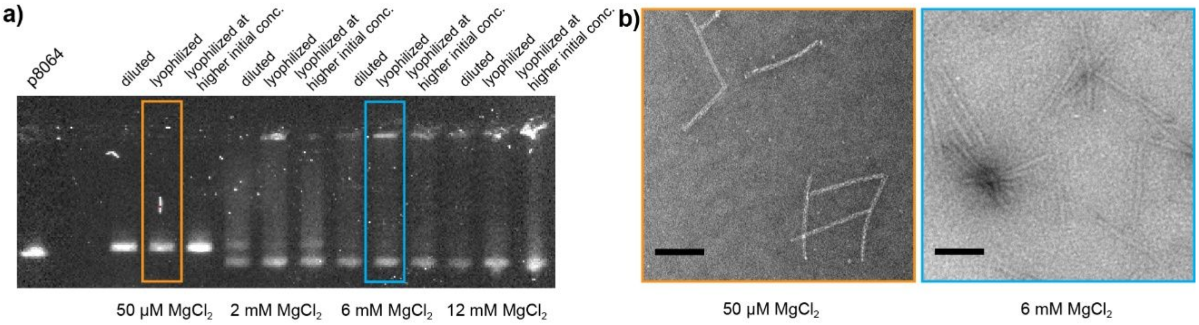

Figure S6: Different $MgCl_2$ concentrations were tested for the lyophilization of DNA origami. (a) Representative agarose gel image for the 14HB. Four different initial buffers with 50 µM, 2 mM, 6 mM and 12 mM $MgCl_2$ were prepared. The samples were diluted to 10 nM or 50 nM final DNA origami concentration in the respective buffer and then lyophilized and afterwards resuspended in ultrapure water. The agarose gel contains a diluted (10 nM) and two lyophilized (one with an initial concentration of 10 nM and one with an initial concentration of 50 nM) samples at final concentrations of 10 nM for each buffer. Samples with a $MgCl_2$ content of 50 – 250 µM exhibit a significantly slower electrophoretic mobility than all samples with a minimum salt concentration of 2 mM $MgCl_2$. This could possibly be attributed to a slight increase in size of the 3D DNA origami nanostructures due to reduced charge screening and an increased repulsion between the negatively charged DNA backbones. A higher initial amount of salt in the sample solution clearly leads to an increased amount of aggregates in the gel wells. (b) Representative TEM images for 14HB samples that were initially dispersed at 10 nM in a solution with 50 µM or 6 mM $MgCl_2$, respectively, and then lyophilized. All samples remained structurally intact during and after the procedure. Scale bars are 100 nm.

Note S3: Stability of different bare 3D DNA origami in low salt buffers

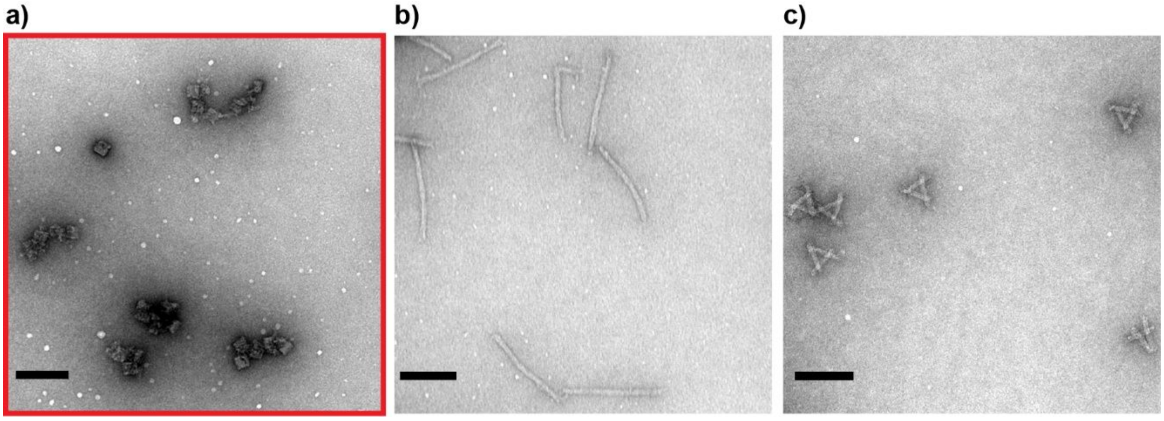

Figure S7: TEM images showing the different behavior/stability of 3D DNA origami nanostructures in very low salt solutions. (a) The cube which is a very compact structure consisting of 112 tightly packed helices disintegrates when dispersed in an aqueous solution containing 500 µM $MgCl_2$. Because of this, a buffer with 2.5 mM $MgCl_2$ was chosen for the lyophilization experiments. Contrary to that, (b) the 14HB and (c) the SC (whose struts are short 14HBs) remain structurally intact even in a buffer with only 50 µM $MgCl_2$. Scale bars are 100 nm.

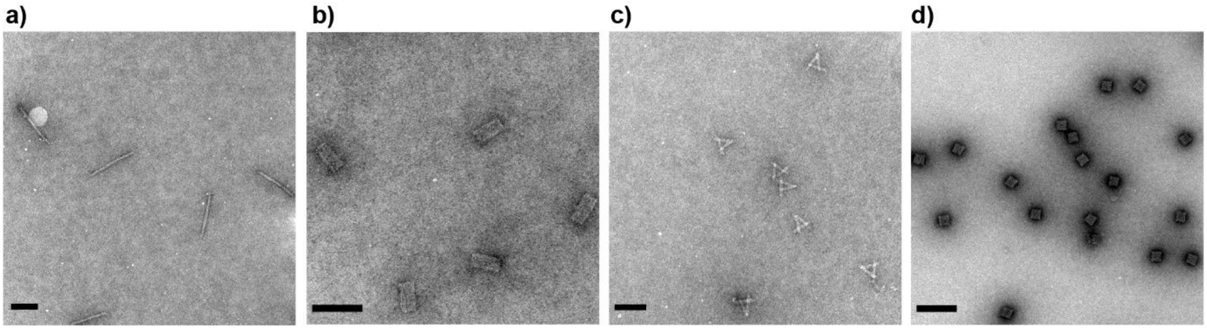

Figure S8: TEM images showing the stability of different 3D DNA origami nanostructures after storage at room temperature for 24 d in a low salt solution: (a) 14HB, (b) 4LB, (c) SC, (d) cube; salt concentrations in (a), (b) and (c): 500 µM MgCl2, (d): 2.5 mM $MgCl_2$. Scale bars are 100 nm.

a) b)

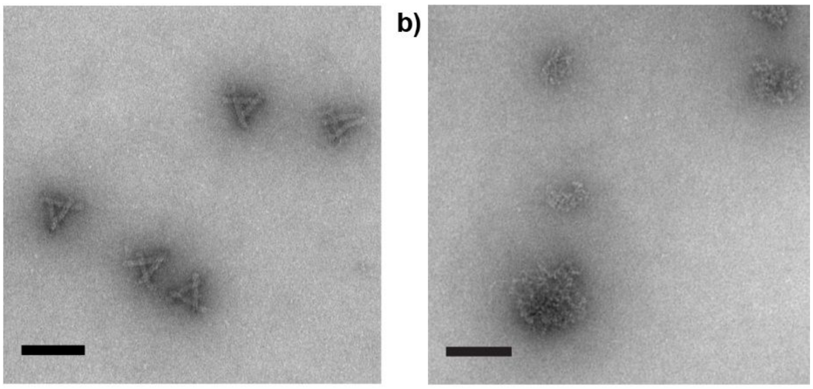

Figure S9: Representative TEM images for DNA origami samples dispersed in buffers with very low salt concentration (50 µM $MgCl_2$). (a) SC structures clearly remain intact when dispersed in a solution containing only 10 mM Tris in addition to the $MgCl_2$. (b) Contrary to that, DNA origami disintegrate quickly when kept in 1 × TA buffer containing the same amount of $MgCl_2$. Scale bars are 100 nm.

Note S4: Storage of lyophilized 3D DNA origami nanostructures

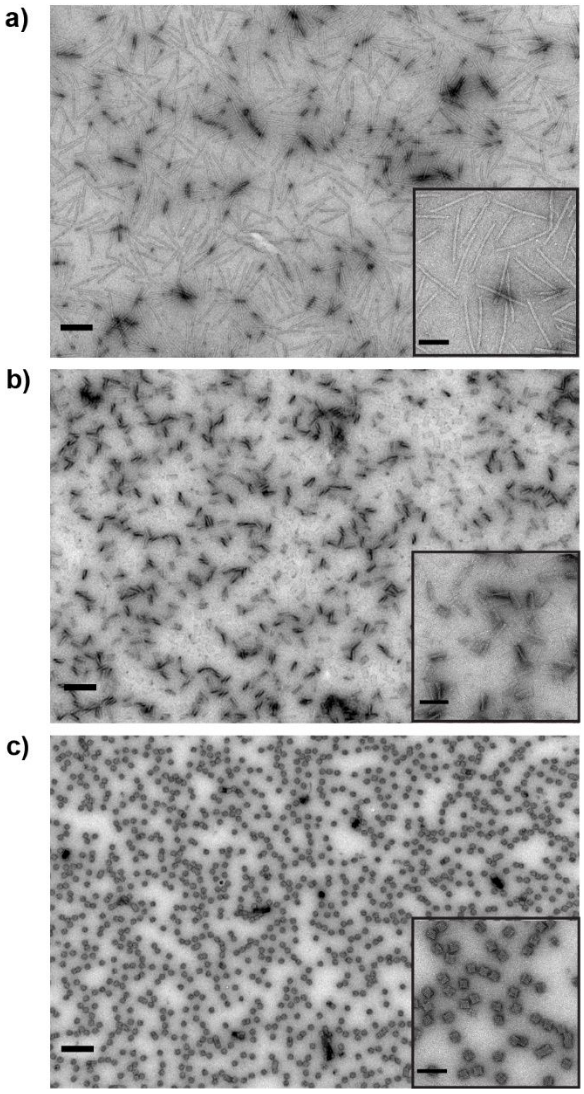

Figure S10: TEM images showing the structural integrity of exemplary 3D DNA origami nanostructures that were stored at room temperature for 5 days after lyophilization (in their freeze-dried state as a white powder) and then resuspended in ultrapure water as described before. (a) 14HB, (b) 4LB, (c) cube. The DNA origami structures exhibit no sign of structural damage and a significantly reduced amount of aggregation, confirming that lyophilized DNA origami samples can be stored in ambient conditions. Scale bars are 200 nm (large images) and 100 nm (insets).

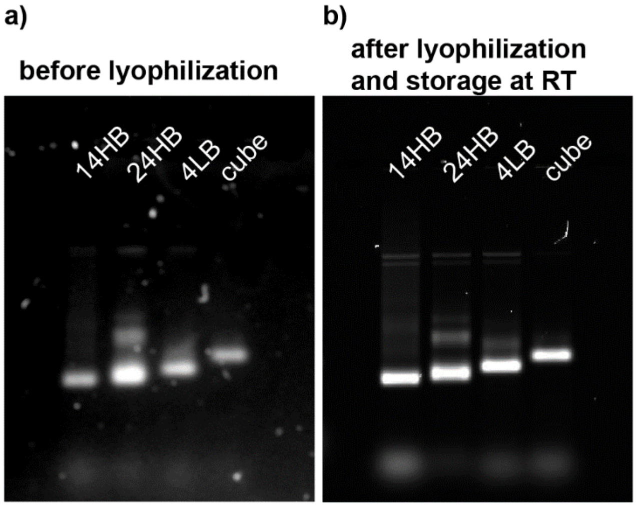

Figure S11: Agarose gel images showing (a) DNA origami nanostructures after purification via PEG precipitation before lyophilization and (b) the same DNA origami nanostructures after storage as a lyophilized powder at ambient conditions for five days and subsequent resuspension in ultrapure water. No significant detrimental effects of lyophilization and subsequent storage could be observed.

Note S5: Summary of important parameters for the lyophilization experiments on 3D DNA origami

**Parameter**	**Recommendation**
Sample volume	Tested up to 750 µL in a 2 ml Eppendorf tube
Choice of tube	Round-bottom tube preferable<br>Additional lid with small holes
Choice of buffer	$MgCl_2$ content: 50 µM – 2.5 mM, depending on the specific DNA origami structure and on the resuspension volume
DNA origami concentration (before lyophilization)	10 – 15 nM
Freezing procedure	Shock-freezing in liquid nitrogen
Resuspension volume (ultrapure water)	Depends on desired final concentration; not lower than 25 µL

Table S1: Overview over important parameters of the lyophilization experiments.

Note S6: Comparison of different methods for purification and concentration of DNA origami

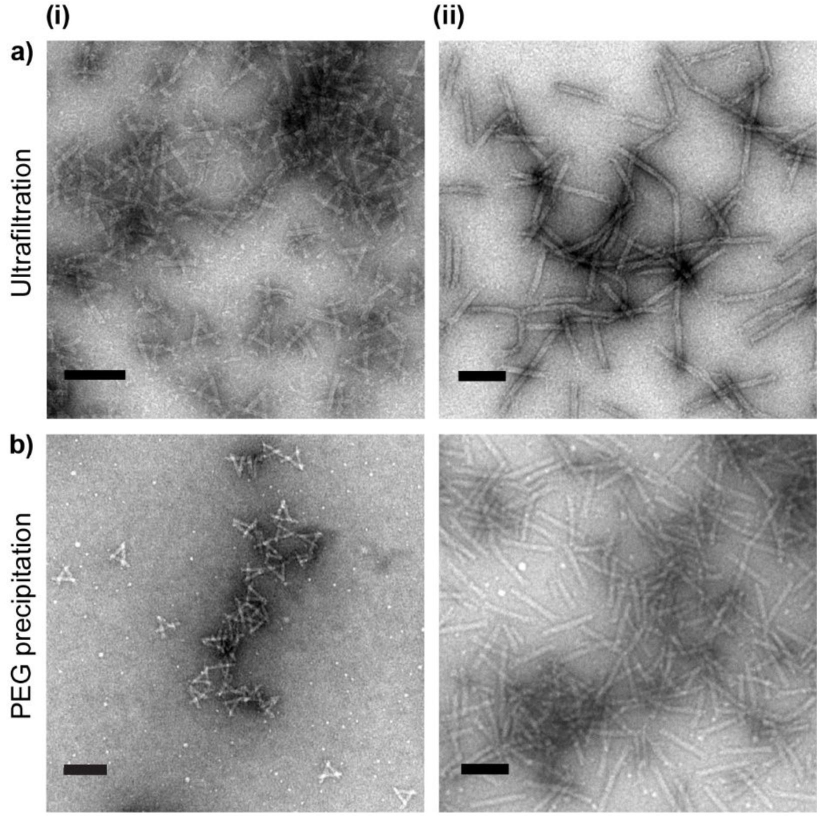

Figure S12: Comparison of purification and concentration of two exemplary 3D DNA origami nanostructures ((i) SC and (ii) 24HB) with (a) ultrafiltration and (b) PEG precipitation. For both methods, a significant amount of aggregates negatively affects the quality of the samples. Scale bars are 100 nm.

Note S7: Different types of aggregation for 3D DNA origami

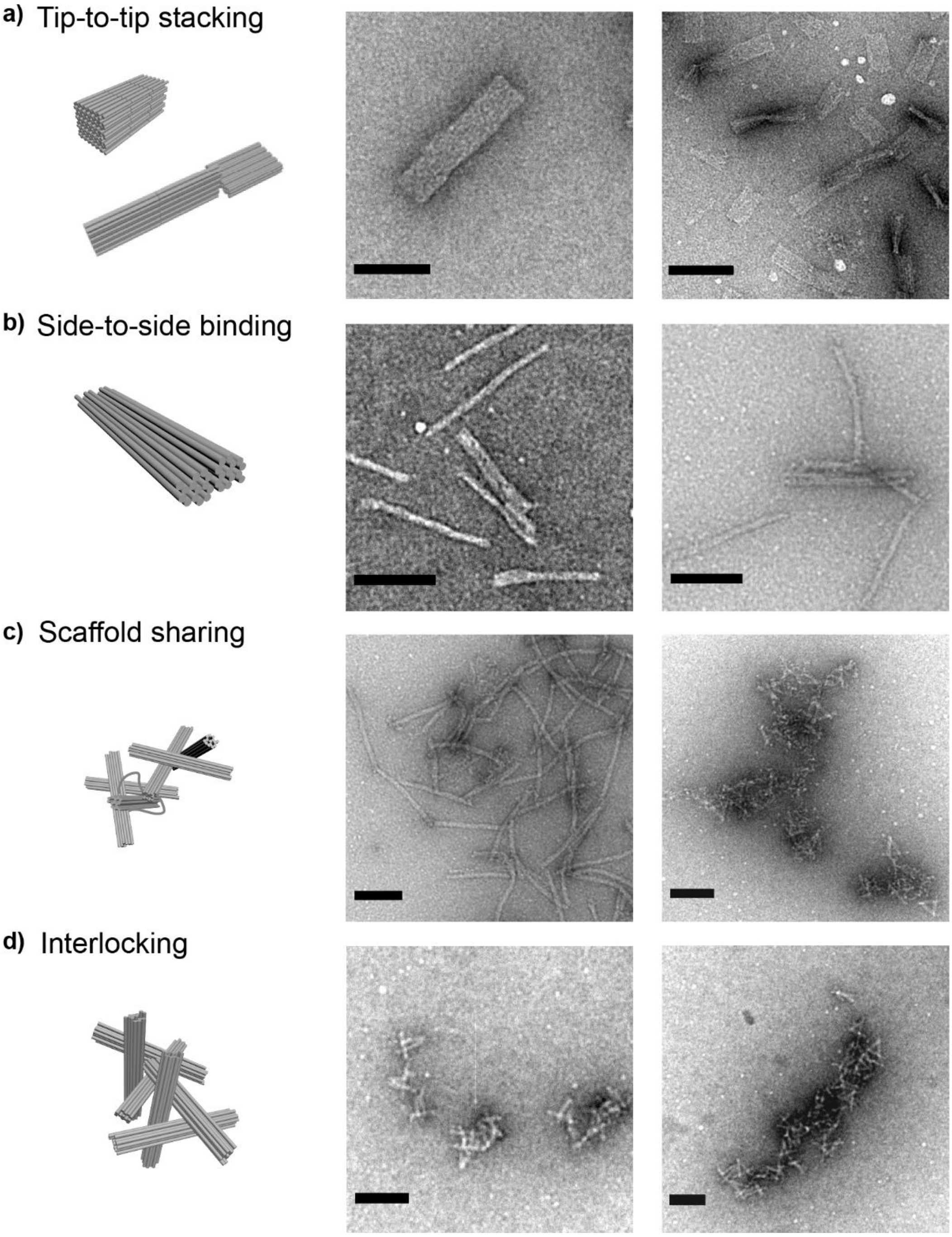

Figure S13: Illustration of a variety of different types of aggregation commonly occurring among 3D DNA origami nanostructures, supplemented with exemplary TEM images. (a) Many rod-like DNA origami such as the 14HB or the 24HB, but also the cube and the 4LB exhibit the tendency to undergo tip-to-tip stacking (here only shown for the cube and the 4LB). Possible design strategies to decrease

such stacking involve the inclusion of single-stranded scaffold loops at the tips of the DNA origami or the addition of poly-T or poly-C tails. Nevertheless, even the implementation of such strategies does not fully eliminate tip-to-tip-stacking in the samples. (b) Some 3D DNA origami (here shown for the 14HB) also undergo side-to-side binding. (c) Defective monomers sharing the same scaffold or misfolded monomers with unfolded scaffold parts can also lead to aggregation due to other monomers getting caught up by the 'loose' scaffold region. The yield of correctly folded monomers can be improved by the optimization of the design and the folding procedure. (d) Especially complex 3D DNA origami can undergo structural interlocking owing to the design. Scale bars are 50 nm (left image in (a)) or 100 nm (all other images).

Note S8: Additional TEM images for different up-concentrated DNA origami nanostructures

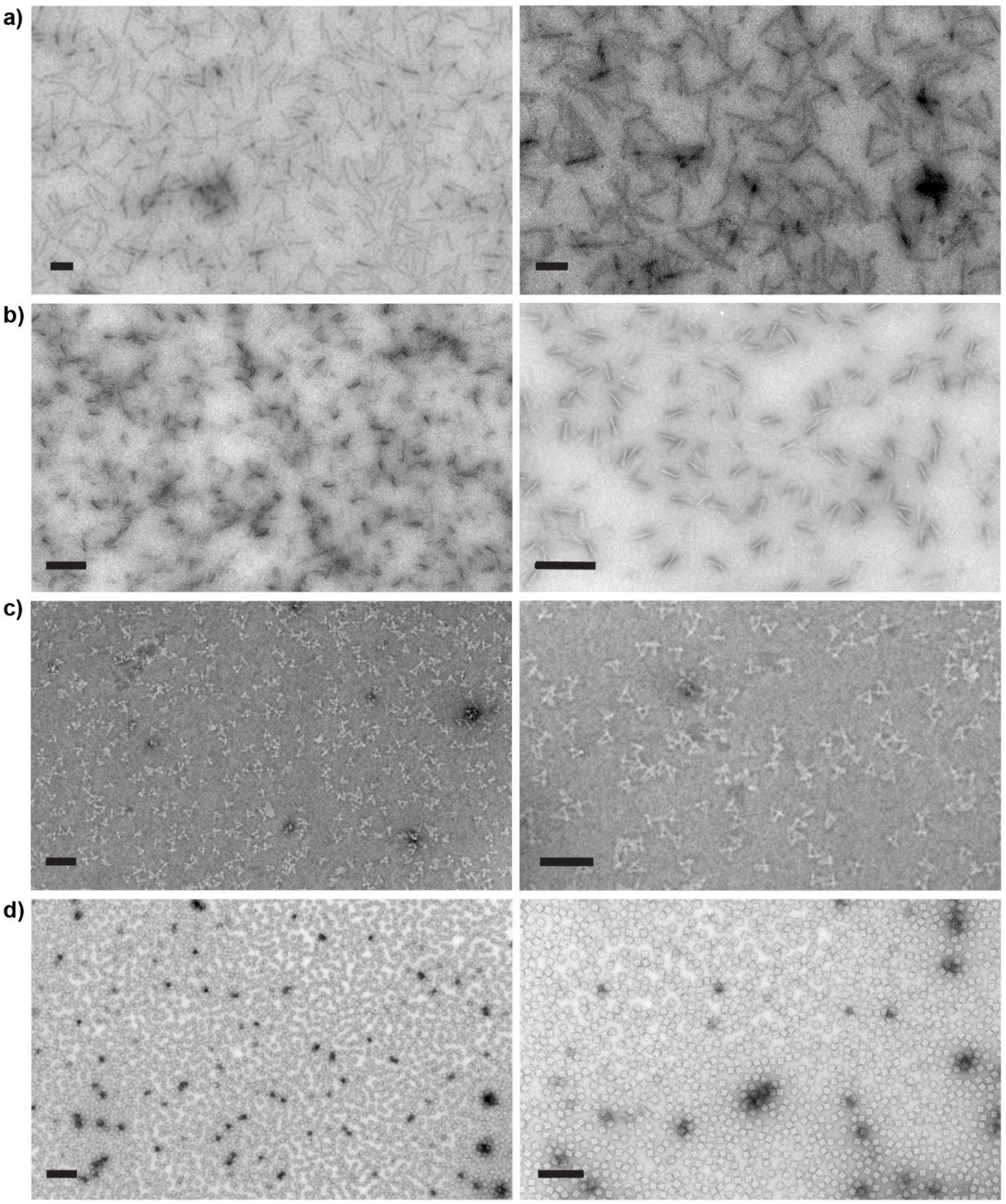

Figure S14: Additional representative TEM images for DNA origami nanostructures which were strongly concentrated (200 – 250 nM) via lyophilization and subsequent resuspension in a low volume of ultrapure water. (a) 14HB, (b) 4LB, (c) SC, (d) cube. Scale bars are 200 nm.

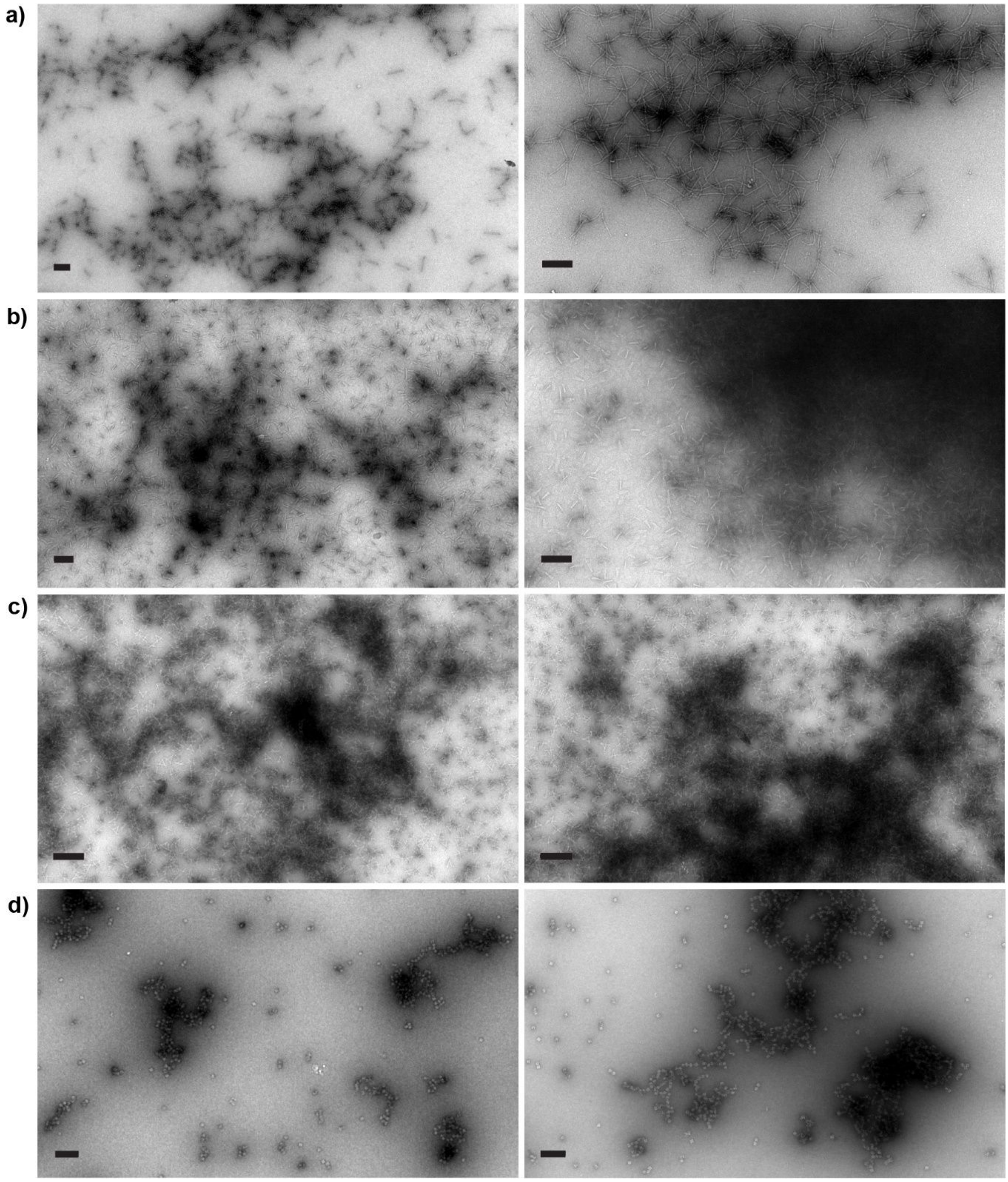

Figure S15: Additional representative TEM images showing different DNA origami nanostructures that were strongly concentrated (200 – 250 nM) via PEG precipitation. (a) 14HB, (b) 4LB, (c) SC, (d) cube. Scale bars are 200 nm.

Note S9: TEM images for silicified DNA origami nanostructures after up-concentration via ultrafiltration

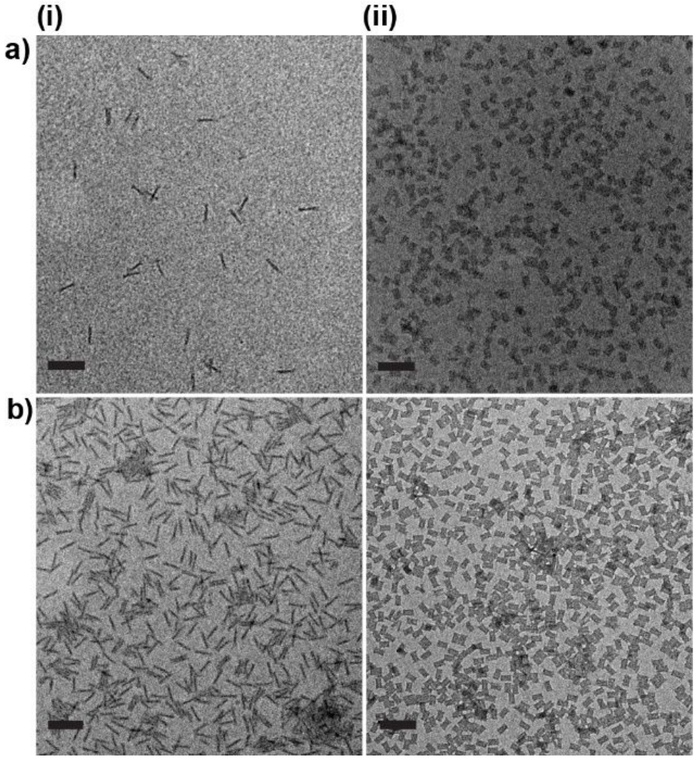

Figure S16: TEM images for silicified DNA origami nanostructures ((i) 24HB and (ii) 4LB) (a) after one round of purification and dilution of the sample and (b) after purification and subsequent up-concentration via ultrafiltration. The handling of silicified DNA origami requires extra caution and up-concentration via ultrafiltration can lead to increased clustering or aggregation. Scale bars are 200 nm.

References

1. Douglas, S. M.; Marblestone, A. H.; Teerapittayanon, S.; Vazquez, A.; Church, G. M.; Shih, W. M., Rapid prototyping of 3D DNA-origami shapes with caDNAno. *Nucleic Acids Res.* **2009,** *37* (15), 5001-5006.
2. Zhang, T.; Hartl, C.; Frank, K.; Heuer-Jungemann, A.; Fischer, S.; Nickels, P. C.; Nickel, B.; Liedl, T., 3D DNA Origami Crystals. *Adv. Mater.* **2018,** *30* (28), 6.
